# Delirium knowledge and assessment by ICU practitioners in South Africa: results of a national survey

**DOI:** 10.1186/cc14562

**Published:** 2015-03-16

**Authors:** S Chetty, F Paruk

**Affiliations:** 1 University of the Witwatersrand, Johannesburg, South Africa

## Introduction

Delirium recognition in critically ill patients is considered to be important taking into account the poor outcomes associated with its occurrence. The purpose of this study was to evaluate knowledge pertaining to delirium as well as the implementation of screening practices. This study constituted a component of a survey that explored current sedation-related practices in South African ICUs.

## Methods

Following approval from the University Human Research ethics committee, a validated questionnaire was distributed electronically to physician members of various medical databases in South Africa as South Africa does not have a formal registry of critical care practitioners.

## Results

One hundred and twenty-six of 174 respondents indicated that they practice in the ICU setting. Sixty-six per cent were specialists and mainly anaesthesiologists (42%), whilst 32% were critical care subspecialists. The respondents indicated that on average 30 ± 20% of their patients experience delirium. Eighty per cent of the respondents indicated that delirium impacts significantly negatively on patient outcomes whilst 1% indicated that there was no such association. Delirium screening is achieved mainly by clinical assessment (77%). Twenty-four per cent utilise an objective tool to screen for delirium and amongst them the CAM-ICU is utilised by 80%. Amongst delirious patients the sedative of choice is dexmedetomidine in the majority. However, 20% prescribe midazolam as a first choice in this setting.

## Conclusion

The findings are comparable with reports of similar surveys conducted in other regions. The delirium screening method is inadequate as the vast majority do not utilise an objective method.

